# Optimization of Film-Dressings Containing Herbal Extracts for Wound Care—A Quality by Design Approach

**DOI:** 10.3390/gels11050322

**Published:** 2025-04-25

**Authors:** Diana Antonia Safta, Cătălina Bogdan, Sonia Iurian, Mirela-Liliana Moldovan

**Affiliations:** 1 Department of Dermopharmacy and Cosmetics, Faculty of Pharmacy, “Iuliu Haţieganu” University of Medicine and Pharmacy, 12 I. Creangă St., 400010 Cluj-Napoca, Romania; diana.an.safta@elearn.umfcluj.ro (D.A.S.); mmoldovan@umfcluj.ro (M.-L.M.); 2 Department 2, Faculty of Nursing and Health Sciences, “Iuliu Haţieganu” University of Medicine and Pharmacy, 4 L. Pasteur St., 400349 Cluj-Napoca, Romania; 3 Department of Pharmaceutical Technology and Biopharmacy, Faculty of Pharmacy, “Iuliu Haţieganu” University of Medicine and Pharmacy, 41 V. Babes St., 400012 Cluj-Napoca, Romania; sonia.iurian@umfcluj.ro

**Keywords:** design of experiments, DoE, QbD, polymeric films, wound healing, film-forming gels

## Abstract

Despite the potential of film dressings for wound healing, many formulations lack an optimized design in order to ensure that the ingredients were carefully chosen to increase the product’s efficacy and stability, while also ensuring the patient’s comfort during the treatment. Moreover, commercially available film dressings do not contain herbal extracts or other active substances with wound healing properties, highlighting a gap in the market and the need for further research in this direction. The aim of this work was the development and optimization of a bio-inspired formulation of a complex herbal extract-loaded film-dressing to be used in wound care, using the quality by design approach. After setting the quality target product profile with the critical quality attributes and undergoing the risk assessment, the design of experiments was implemented. All the selected ingredients were biodegradable, aligning with the current need for a natural approach, based on their biocompatibility and reduced environmental impact. A D-optimal experimental plan was used, in which the types and concentrations of film-forming agents and plasticizers were varied: xanthan gum, acacia gum, sodium carboxymethylcellulose and glycerol, 1,3-propanediol, and xylitol, respectively. All formulations contained polyvinyl alcohol and a previously studied complex herbal extract. The films were characterized in terms of uniformity of mass, film thickness, swelling degree, folding endurance, adhesive, and mechanical properties. The optimized formulation was achieved by maximizing the swelling degree, adhesive properties, hardness, deformation at target, and elongation at break. The optimized film was characterized, and the in vitro total polyphenolic content release from the film was evaluated. Following the understanding of the influences of the formulation factors on the film characteristics, the composition of the optimized film-dressing was determined as follows: 5% polyvinyl alcohol, 0.25% xanthan gum, 10% glycerol, and 20% complex herbal extract. The optimized film exhibited high swelling degree (627.28%), high adhesive properties (adhesive force of 28.00 g and adhesiveness of 0.20 mJ), high elasticity (deformation at target of 29.80%, and elongation at break of 106.90%), as well as good mechanical properties (hardness of 2616.00 g), which are suitable characteristics for use on wounds. Moreover, the optimized film-dressing exhibited a sustained release, with a maximum release of polyphenols of 88.00% after 8 h.

## 1. Introduction

The growing prevalence of wounds of different etiologies has become a critical healthcare challenge in recent years [1,2,3]. Wound healing is a complex process driven by interconnected pathways that facilitate tissue repair and regeneration. This process involves various events, such as the migration of inflammatory cells, cytokine activity, collagen deposition, extracellular matrix formation, and scar tissue remodeling, resulting in scar formation [2,4].

Given the wide-ranging characteristics of wounds, including their type, severity, and location, wound dressings are highly diverse, varying regarding material composition, shape, structure, and functional properties. Currently, research in the field of topical systems for wound healing is focused on the development of modern dressings, like film-dressings. Their major advantages include the ability to absorb excess inflammatory exudate, maintain a moist environment in the wound, a critical component of effective healing, and be permeable to water vapor and air but impermeable to bacteria or other contaminants [5,6,7]. The use of modern dressings can be the easiest, most accessible, and most cost-effective way to treat wounds and, hence, holds significant promise [8].

Film-dressings are thin, flexible materials composed of a polymer layer, usually with an added plasticizer. Due to their flexibility, film-dressings are perceived to be more comfortable by patients, acting as drug-delivery platforms with non-invasive properties that enhance drug action onset and efficacy [9]. Film-dressings exhibit excellent mechanical properties, particularly elasticity and flexibility, enabling them to adapt and conform to a wide range of wound shapes. The transparency of polymer films facilitates wound monitoring without the need for dressing removal [10]. In addition to this, effective wound dressing favors healing by protecting the wound from external factors while allowing oxygen exchange, managing bacterial load, maintaining optimal moisture levels, and protecting the incorporated active ingredients from degradation [11]. Moreover, the possibility to incorporate wound healing substances in film-dressings has opened a whole new world of possibilities [5,6,7].

In line with these features, recent research has concentrated on developing innovative, safe, and cost-effective topical systems that incorporate herbal extracts or phytocompounds in products that offer complex beneficial effects for skin health [2,12]. The integration of herbal extracts into polymer-based wound dressings, such as films, has been shown to improve therapeutic outcomes, including enhanced antibacterial, antioxidant, and wound healing activities [10,13,14]. For example, Colobatiu et al. developed film- dressings loading *Plantago lanceolata*, *Arnica montana*, *Tagetes patula*, *Symphytum officinale*, *Calendula officinalis*, and *Geum urbanum* extracts, using quality by design (QbD) to optimize the formulation and to study the effects of different excipients on the films’ properties [14]. In another study, an optimized film-dressing with herbal extracts was evaluated as a wound dressing with both in vitro and in vivo promising results in acceleration of the wound healing process [13]. Jridi et al. developed a film-dressing loading a *Lawsonia inermis* aqueous extract which showed increased wound healing activity and prevention from inflammation damage, increased antioxidant enzymatic activities, and decreased malondialdehyde compared to control and a commercially available product [15]. Another successful development of herbal extract-loaded film-dressings was presented in the paper published by Chin et al., incorporating *Moringa oleifera* standardized extract in film-dressings with adequate physicochemical properties [16].

In addition to the great benefits of the use of herbal extracts in wound care, it is well-known that the polyphenols from herbal extracts are susceptible to factors such as temperature, light, oxygen, and metal ions, which affect their biological activity, especially the antioxidant potential, greatly limiting their bioavailability. A favorable approach involves the inclusion of the extracts or the isolated polyphenols in sustained-release films to improve polyphenol utilization [17]. Moreover, using film-dressings can also increase their bioavailability by producing skin occlusion and enhanced contact with the skin surface [18]. Although most of the research has been carried out with isolated compounds from herbal extracts, the combination of phenolic compounds in the whole extract can create a synergism between them and enhance their activity [19]. This strategy was applied to the current study, which incorporated in film-dressings a complex herbal extract (CHE) that was previously optimized using the QbD approach. The CHE consisted of equal parts of three hydroethanolic optimized extracts obtained by Ultra-Turrax-assisted extraction, from *Sambucus nigra* flowers, *Epilobium hirsutum* aerial parts, and *Lythrum salicaria* aerial parts. Each of the three extracts were optimized by maximization of the total polyphenolic content and total flavonoid content, that were found to be important agents in the wound healing process. Their rich phytochemical composition led to the demonstrated complementary biological activities, such as wound healing, anti-inflammatory, antioxidant, and antimicrobial effects, that are essential in wound care [20].

The aim of this study was to optimize a film-dressing formulation, using the QbD approach, to address the wound care challenge with improved performance and functionality. Employing the design of experiments (DoE) method, a valuable tool of the QbD approach, the impact of the different types and concentrations of excipients, specifically film-forming agents (xanthan gum, acacia gum, sodium carboxymethylcellulose) and plasticizers (glycerol, 1,3-propanediol, xylitol), on the quality and performance of the dressings was thoroughly investigated. These excipients were carefully chosen to contribute to the wound healing effect of the incorporated CHE and to enhance the quality characteristics of the films. Moreover, their effects on the film dressings’ characteristics have yet to be extensively studied. Thus, the results of their influences can be valuable for other researchers to design their films for various applications. The optimized formulation was developed based on the first established quality target product profile (QTPP) and the observed effects of formulation factors on the critical quality attributes (CQAs). Thus, the swelling degree, mechanical, and adhesive properties were maximized, to increase the film’s efficacy as well as the patient’s comfort during the treatment. Subsequently, the optimized CHE-loaded film-dressing represents a promising candidate for wound care applications. Further investigations on the optimized CHE-loaded film-dressing are considered, such as physical and thermal stability, comparison with blank film and commercially available film, and in vitro and in vivo evaluations.

## 2. Results and Discussion

### 2.1. Preparation of the CHE-Loaded Film-Dressings

The films obtained presented favorable organoleptic characteristics, having a light-yellow color, due to the color of the CHE. All films were transparent, homogeneous, and had a smooth surface; however, after a period of time, an opacification of xylitol containing films was observed. The literature reports that films prepared using the solvent casting method tend to become brittle while stored, as evidenced by a reduction in the film’s percentage of elongation over time because of evaporation or loss of the residual solvent [9]. However, by modulating the formulation parameters, films with suitable characteristics were successfully obtained.

### 2.2. The QbD Approach

A systematic approach to film-dressing development is crucial to a better understanding of their effects on the mechanical, adhesive, and swelling properties of the films. This can be achieved by using the QbD approach with the DoE tool. In this framework, the formulation factors were selected from among the most promising substances that have not yet been subjected to the DoE method; thus, their combination requires comprehensive investigation. All the chosen ingredients were biocompatible and biodegradable.

The QbD approach represented a paradigm shift in pharmaceutical development, emphasizing a systematic, science-based methodology to ensure robust and reproducible formulations, modeling and controlling the manufacturing process itself, not just relying on quality tests at the end of the process [21,22,23]. In the context of film-dressings for wound healing, QbD enables the identification and control of the critical material characteristics (CMCs) and critical process parameters (CPPs) early in the development cycle. The integration of the DoE within this framework provides a structured strategy for studying multiple factors simultaneously, offering insights into their interactions and impacts on the formulation’s performance. By observing the variables’ influences through statistical modeling, the DoE can optimize the formulation ensuring the desired qualities (from the previously established QTPP) and ultimately enhances the therapeutic efficacy, safety, and simplifying the use of wound-healing films. Furthermore, the systematic nature of QbD minimizes variability between production batches, ensuring consistent safety and efficacy in further applications, ultimately meeting both patient needs and regulatory standards [14,24,25,26].

### 2.3. Establishment of the QTPP and CQAs of the Film-Dressings

Within this study, the QTPP of CHE-loaded film-dressing was built to obtain a formulation with potential high effectiveness and overall performance, with a particular interest in increasing the probability to assure the future patients’ comfort during the treatment (Table 1).

The first CQA was the swelling degree of the film-dressings. Adequate swelling creates a moist environment, which is critical for effective wound healing, while also ensuring that excess fluid is absorbed without leading to skin maceration. This helps in autolytic debridement and promotes faster tissue regeneration [27,28]. It has been demonstrated that a wound dressing with a higher moisture absorption rate heals more quickly than one with a lower rate, leading to the choice to maximize the swelling degree of the optimized films [29]. Moreover, the jellification of the film after swelling facilitates the release of active ingredients from the CHE, where an increased swelling degree corresponds to prolonged drug release. The swelling behavior is attributed to the ionization of hydroxyl groups in the polymeric network [30].

The adequate mechanical strength (hardness) of the films was considered the second CQA, to withstand handling, application, and environmental stress without tearing or breaking. This ensures the wound remains protected from contaminants, physical damage, and additional trauma, providing structural support to the wound area, enabling easy handling, maintaining good integrity during storage and application to the lesion [7,31]. Another goal was to ensure the films had a high degree of elasticity, given by the maximization of deformation at target and elongation at break, leading to films both flexible and resistant to ruptures, and allowing them to stretch and adapt to the wound site and the body’s movements to provide a snug fit. Elasticity keeps the film from becoming overly rigid, which could irritate the wound or harm the skin around it, and enables the film to adjust to swelling, motion, and variations in the size of the wound [7,31,32]. Wound dressings should possess sufficient mechanical strength to withstand handling and application while maintaining adequate flexibility for patient comfort.

Additionally, good adhesive properties were established as another CQA to ensure that the film stays in place for the required duration of usage, maintaining an optimal healing environment and providing continuous protection. Adequate adhesion helps seal the wound, preventing the entry of pathogens and contaminants, which reduces the risk of infection. Appropriate adhesive properties ensure that the film can be applied easily and then removed without causing trauma to the wound or surrounding skin [7,33]. The combination of high flexibility and adhesive properties enable the film to conform to the shape of the body where it is applied, allowing movement without pain or harm.

To summarize, all these CQAs and their target values were chosen to improve the physical resistance of the film-dressing, the maintenance of the moist environment at the wound site, the patient comfort during the treatment, and to support the wound healing process, as detailed in Table 1.

**Table 1:** Quality target product profile for CHE-loaded film-dressing.

QTPP			
Parameter	Importance	CQAs	Target
Appearance	Visual inspection of the wound	-	Transparent and homogenous, CHE-colored [14]
Application	Appropriate topical application to the wound	-	Intimate contact of the film with the wound site [14]
Uniformity of mass (g)	Homogenous quantity of phytocompounds from CHE applied to the skin	-	Average weight ±10% [34]
Thickness (mm)	Application ease, flexibility, and film aesthetics	-	0.10–0.20 [14,24,35]
Swelling degree (%)	Absorbance of the exudate from the wound and jellification	Yes	Maximized [7,29]
WCA (°) *	Jellification of a hydrophilic surface	-	<90° [36]
pH *	Skin compatibility, wound healing promotion	-	4.1–5.8 [37,38]
WVTR (g/m^2^/day) *	Appropriate permeation of gases and water	-	2000–2500 g/m^2^/day [36]
Folding endurance	Flexibility during storage and usage to avoid cracks or breakage	-	Resistance to >300 folds without visible cracks [9,39]
Hardness (g)	Resistance during storage and application to avoid cracks or breakage	Yes	Maximized >2500 g [24]
Rigidity at 5 mm (g) (hardness at 5 mm)	Resistance during storage and application to avoid cracks or breakage	-	High values [40]
Deformation at target (mm)	Conformability to irregular or moving surfaces of the skin, comfortable during usage	Yes	Maximized [31]
Adhesiveness (mJ)	Appropriate adhesion and covering to protect the wound side, contact with wound site, and the release of the CHE active ingredients	Yes	Maximized [7]
Adhesive force (g)	Appropriate adhesion and covering to protect the wound side	Yes	Maximized [7]
Tensile strength (MPa)	Resistance during storage and application to avoid cracks or breakage	-	1–32 MPa [41,42]
Elongation at break (%)	Flexibility during storage and usage to avoid cracks or breakage, comfortability during usage	Yes	Maximized [31]
Young’s modulus (MPa)	Conformability to irregular or moving surfaces of the skin, comfortability during usage	-	0.4–20 MPa [42,43]
In vitro active substances release	Effectiveness of the treatment, reduced frequency of dressing changing	-	Sustained release for at least 6 h [24]

* these characterization methods will be further included in another paper that is currently in preparation. WCA—water contact angle, WVTR—water vapor transmission rate.

### 2.4. Risk Analysis

The Ishikawa diagram (Figure S1) was first utilized to map out and organize potential sources of variation in the formulation process, focusing on the key factors influencing the CQAs. This has helped in identifying the potential root causes of defects and inconsistencies. Then, the FMEA (Table S1) provided a comprehensive approach to assessing the potential failure modes at each stage of formulation and production, facilitating the proactive management of risks and ensuring that the final product meets the defined QTPP. The combination of these methods allowed for a thorough and systematic risk assessment to optimize the product development process, our objective being a reduction of the risk of failure and an evaluation of the effects of modifying the most important variation factors on the quality of the films. As observed from Table S1, the highest RPN values were found for the types and ratios of the film-forming agents and plasticizers, indicating their critical role in determining film performance. Therefore, the selection of these components is a key factor in the development and optimization process and, as such, these variables were incorporated into the DoE.

### 2.5. Development of the DoE

Following the risk analysis, the DoE was developed to optimize the formulation of the film-dressings by systematically evaluating the impact of various process and formulation variables on the CQAs. Thus, in the current study, three co-film-forming agents, namely acacia gum, xanthan gum, and sodium carboxymethylcellulose (CMC-Na) were selected to study the characteristics of the films when associated with polyvinyl alcohol (PVA) in different ratios. The incorporation of these co-film-forming agents was hypothesized to improve the properties of the PVA-based films, compensating for potential limitations of pure PVA, such as excessive brittleness or suboptimal swelling [44]. The effects of different types and ratios of three plasticizers, glycerol, 1,3-propanediol, and xylitol were also investigated. A D-optimal design was implemented to analyze the interactive effects of the variables on the product’s performance. The CHE concentration was maintained at a fixed level across all formulations in the DoE (20% *w*/*w* from the total mass of the film) to evaluate the effects of the formulation variables related to the excipients.

PVA was selected as the principal film-forming agent, based on its good film-forming properties, in a constant concentration that was determined through preliminary formulations (5% *w*/*w* from the total mass of the film). PVA is a hydrophilic polymer used in the fabrication of films, implants, and hydrogels, among many more [45,46]. PVA is one of the preferred achievable ingredients to produce composites, thanks to its biodegradability and good mechanical properties, keeping also its flexible and adhesive properties [47,48]. Previous studies have shown that the copolymerization of PVA with other film-forming polymers formed composites with improved properties as wound dressings [14,28,45,49,50,51].

Consequently, three other film-forming agents, both synthetic and natural, were selected, and the effects of their association with PVA were analyzed. As synthetic co-film-forming agent, CMC-Na was chosen. CMC-Na is a tissue resembling polymer, forming promising films for antimicrobial and wound dressing applications [52,53]. CMC-Na is a reinforcement material in composites of polymer matrix, enhancing the mechanical, barrier, and thermal properties of the film [52]. Previously, films with CMC-Na were studied for their antimicrobial and wound healing effects with promising results [53]. Moreover, CMC-Na gauze is commercially available [52,54]. The combination of synthetic and natural polymers to develop biomaterials has emerged as a significant research field, by enhancing their properties and functionalities [55]. Thus, two gums were chosen as natural polymers to be associated with PVA. Gums, as a group of polysaccharides, are hydrocolloids and water-soluble biopolymers, which can be mixed with synthetic polymers to form films [56]. Among them, xanthan gum, a natural polysaccharide obtained from *Xanthomonas campestris* (*Xanthomonadaceae* family), has gained attention in recent years to be used in wound care systems [57,58,59]. Xanthan gum has high viscosity at low concentrations [60], and higher thermal stability than other polysaccharides [58]. On the other hand, acacia gum (gum arabic) is one of the oldest and the most widely used gums [61], containing a mixture of polysaccharides and glycoproteins [62]. Acacia gum is an exudation obtained from the stems and branches of *Acacia senegal* or closely related species of *Acacia* sp. (*Leguminosae* family) [33]. It was used as a demulcent with antioxidant, anti-inflammatory, and antibacterial effects which can aid in the wound healing process [63,64]. It has also been reported to exhibit hemostatic properties due to its anionic character that can induce the coagulation of blood [33,65]. Recently, acacia gum was mixed with PVA to form nanofibers or films with good results [48,56].

Apart from the film-forming agents, film-dressings usually contain plasticizers, like polyols. In general, plasticizers enhance the flexibility in the polymer structure by reducing intermolecular forces, which increases the mobility of the polymer chains in the film matrix [66,67]. Three polyols were chosen as plasticizers to be investigated through the DoE. The first one was glycerol, which is the most widely used hydrophilic plasticizer for films and a well-known skin humectant, serving a dual biological and functional purpose [68,69]. Glycerol may help maintain and preserve the epidermal barrier, with improved wound healing [70]. According to the clinical research, a high concentration of glycerol produces a bacteriostatic environment that reduces the quantity of microorganisms in the wound. Regarding wound treatment, this is unquestionably advantageous and leads to improved healing results [71]. Another polyol investigated was 1,3-propanediol, a natural alternative to propylene glycol as a common solvent, humectant, emollient, and plasticizer [72]. Propylene glycol (1,2-propanediol) is frequently used in the development of polymeric films for its plasticizer effects [73], but due to its potential risk of causing allergic reactions and irritation [74], replacing it with 1,3-propanediol may be a safer choice [75]. The third polyol was xylitol, that has previously been used as plasticizer to replace glycerol [76]. Moreover, it has been demonstrated that xylitol can inhibit bacterial biofilm formation, which can delay wound healing and cause chronic wounds [29]. Xylitol has been used in combination with PVA to form food packaging films [77], but its potential in film-dressings formulations remains underexplored.

The theoretical model applied to each response (Y1–Y10) is presented below. Table S2 presents the revised quantitative factor effects and the associated *p*-values for each response (Y1–Y10).

Y = a0 + a1X1 (AG) + a2X1 (XG) + a3X1 (CMCNa) + a4X2(G) + a5X2(X) + a6X2(P) + a7X3+ a8X4(1)

where a0–a8 represent the regression equation coefficients that show the effect of each factor, Y represents each response (Y1–Y10), X1–X4 are the individual effects (X1—type of co-film-forming agent, X2—type of plasticizer, X3—concentration of co-film-forming agent, X4—concentration of plasticizer, AG—acacia gum, XG—xanthan gum, CMCNa—sodium carboxymethylcellulose, G—glycerol, X—xylitol, P—1,3-propanediol).

### 2.6. Statistical Analysis

Table 2 presents the statistical parameters for ANOVA test and quality of fit in the case of the developed DoE. Moreover, descriptive statistics and more statistical parameters of the model are presented in Tables S3 and S4, respectively.

The results showed that R^2^ values ranged between 0.525 and 0.885, while the Q^2^ values were between 0.313 and 0,753. The model validity values varied between 0.109 and 0.794 and reproducibility between 0.655 and 0.985. A good model is shown by high values for these two parameters. High values of the model validity and reproducibility show a high significance of the chosen model [25,26]. Table 2 shows that all the *p*-values were below 0.05, while the lack of fit values were above 0.05 in most cases (excepting Y6). These values indicated statistically significant models.

### 2.7. Characterization of the CHE-Loaded Film-Dressings

#### 2.7.1. Organoleptic Analysis

The films obtained presented favorable organoleptic characteristics, having a light-yellow color, attributed to the CHE. All films were transparent, homogeneous, and had a smooth surface; however, opacification of xylitol films was observed over time. Transparency of the films is an important feature as it allows for monitoring the evolution of the lesion during treatment, without necessarily removing the dressing. Homogeneity ensures uniform distribution of the CHE, while the smooth surface and flexibility are features that contribute to patient comfort during film application [10,24].

#### 2.7.2. Uniformity of Mass

The uniformity of mass of the film-dressings was assessed to ensure consistency in weight, which directly impacts the uniform distribution of the bioactive ingredients and the overall performance of the dressing. The average mass for each formulation was within ±5% of the mean (standard deviation <±0.005), as presented in Table 3, thus corresponding to the pharmacopeial requirements (average mass ±10%) [34].

#### 2.7.3. Characterization of the CHE-Loaded Film-Dressings: Results from DoE

**Figure 1:**
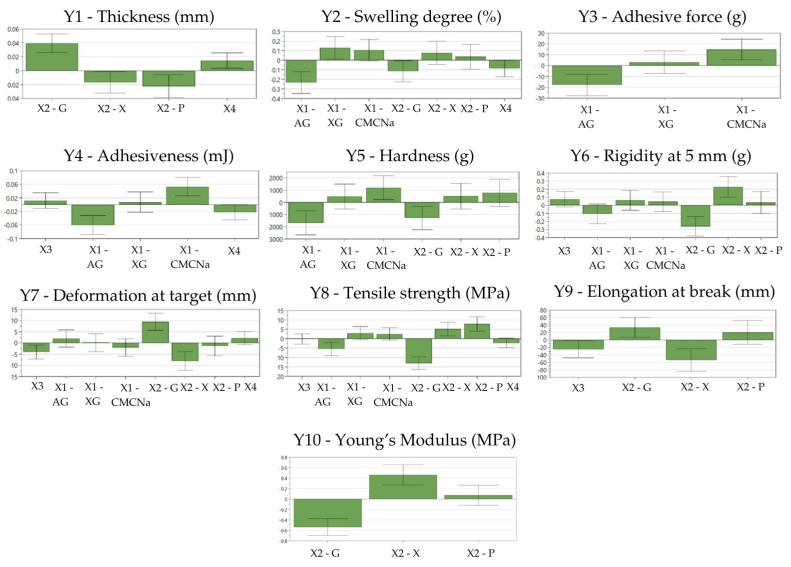
Coefficients plots showing the influences of selected factors on the CHE-loaded film-dressings’ properties AG—acacia gum, XG—xanthan gum, CMC-Na—sodium carboxymethylcellulose, G—glycerol, X—xylitol, P—1,3-propanediol, X1—type of co-film-forming agent, X2—type of plasticizer, X3—concentration of co-film-forming agent, X4—concentration of plasticizer, Y1—film thickness (mm), Y2—swelling degree (%), Y3—adhesive force (g), Y4—adhesiveness (mJ), Y5—hardness (g), Y6—rigidity at 5 mm (g), Y7—deformation at target (mm), Y8—tensile strength (MPa), Y9—elongation at break (%), Y10—Young’s modulus (MPa).

Table 4 presents the DoE and the matrix of the results of characterization of the CHE-loaded film-dressings, and Figure 1 illustrates the coefficient plots. Figure S2 shows the replicate plots, where a random distribution of the results for all the experiments and a small variability of the replicates was observed, suggesting a high impact of the chosen independent variables. For a better understanding of the influences of the formulation factors on the responses, the contour 4D plots are presented in Figure S3.

*The influence of the formulation factors on the thickness of the CHE-loaded film-dressings (Y1)*

Film thickness (Y1) ranged from 0.102 mm (N12) to 0.208 mm (N2), as observed from Table 4. Glycerol increased the film thickness, while xylitol and 1,3-propanediol had the opposite effect (Figure 1). A higher plasticizer ratio generally resulted in thicker films. This observation aligns with previous findings regarding glycerol’s positive influence on film thickness, which is attributed to its intercalation within the polymer matrix [6,78].

*The influence of the formulation factors on the swelling degree of the CHE-loaded film-dressings (Y2)*

The swelling degree (Y2) of the films varied between 446.61% (N2) and 3560.62% (N9), as presented in Table 4. The incorporation of xanthan gum increased the swelling degree, while the addition of glycerol or acacia gum had the opposite effect (Figure 1). This observation aligns with the previous research demonstrating that glycerol, despite its hygroscopic nature, can delay water absorption by films, even though glycerol’s hygroscopic qualities are expected to increase the swelling degree of the film [29]. This behavior is probably influenced by glycerol’s capacity to form hydrogen bonds with water molecules due to the numerous anionic groups present in the molecules’ side chains [79]. Acacia gum decreased the swelling degree, possibly by its intercalation in the PVA polymeric matrix, decreasing the voids in the matrix where the water can be absorbed. Generally, adequate film swelling is important to ensure a moist environment that promotes the rapid migration of cells involved in wound healing [7,33].

*The influence of the formulation factors on the adhesive properties of the CHE-loaded film-dressings (Y3*, *Y4)*

The adhesive force (Y3) of the films varied between 0.15 g (N7) and 0.36 g (N6), while the adhesiveness (Y4) ranged between 13.00 mJ (N7) and 84.80 mJ (N5), as seen in Table 4. The main film-forming agent, PVA, usually ensures the appropriate adhesive properties [47], but the adhesive force and the adhesiveness were significantly influenced by the co-film-forming agents. CMC-Na had a positive impact on adhesion, while acacia gum had a negative influence at the tested concentrations (Figure 1). The increased adhesivity of the film-dressings containing CMC-Na can be attributed to the formation of the hydrogen bonds and other intermolecular forces between the CMC-Na and PVA molecules in the film matrix. These interactions can create a more cohesive film structure, improving adhesion to substrates, as was also observed by Helmiyati et al. [80]. Similarly, excellent adhesive properties were observed by Chang et al. when testing CMC-Na-based hydrogels [81]. By contrast, the use of acacia gum negatively impacted on the adhesive properties of the film-dressings. Generally, natural gums show weak bioadhesive properties [33] and, in this case, acacia gum appears to decrease the adhesive property of the PVA.

*The influence of the formulation factors on the mechanical properties of the CHE-loaded film-dressings (Y5–Y10)*

The hardness (Y5) of the films ranged from 2485.00 g (N2) to 8543.00 g (N11), as observed in Table 4. The main film-forming agent, PVA, ensured the appropriate mechanical properties of all the film-dressings from the experimental plan [48]. Although, the co-film-forming agents and plasticizers significantly influenced the mechanical properties of the films. The hardness of the films was increased by the inclusion of CMC-Na, while it was reduced by using acacia gum and glycerol (Figure 1). The hydroxyl groups present in the structure of both PVA and CMC-Na can explain the formation of inter- and intramolecular hydrogen bonds that contribute to the structural integrity and cohesion of the film matrix, influencing its mechanical properties [82]. The influence of acacia gum and glycerol may be attributed to their intercalation in the polymeric matrix, decreasing the mechanical strength of the film-dressings [78]. In general, plasticizers cause an increase in flexibility in the polymer structure by intercalation in the polymeric matrix, reducing the intermolecular forces, which increases the mobility of the polymer chains in the films [66,78]. Thus, glycerol, being a small molecule, can easily insert into the adjacent polymer chains and increase the flexibility of the films, while decreasing their rigidity. As a result, it improves the handling properties and minimizes the risk of cracking, making it one of the most used plasticizers. [83]. The rigidity at 5 mm, which represented the hardness value registered at 5 mm, (Y6) varied between 669.00 g (N2) and 3203.00 g (N9), as shown in Table 4. The rigidity at 5 mm was decreased by using glycerol, as in the case of hardness, and was increased by using xylitol (Figure 1), a fact that can relate to the crystallization of xylitol observed over time in the films in the tested concentrations. Other researchers also observed that a crystallization phenomenon may occur in films plasticized with xylitol during long-term storage [84]. The deformation at target (Y7) values varied between 7.8 mm (N10) and 35.99 mm (N5), as presented in Table 4. This parameter was positively influenced by the addition of glycerol and negatively impacted by the addition of xylitol and a higher ratio of co-film-forming agent (Figure 1). Xylitol decreased the deformation capacity due to the observed crystallization process, while glycerol increased the deformation due to the plasticizing effects explained above, increasing the flexibility and elasticity of the films [6,85]. A higher ratio of the co-film-forming agent increased the number of cross-links between the polymer chains, which restricted their mobility and resulted in a stiffer and more rigid material with reduced deformation capacity [80,86]. The tensile strength (Y8) values of the films varied between 4.58 MPa (N2) and 34.4 MPa (N14), according to Table 4. The tensile strength was increased by using xylitol or 1,3-propanediol (Figure 1). Xylitol increased the tensile strength because of the crystallization process that occurred, as previously discussed. The polyol 1,3-propanediol increased tensile strength due to the formation of strong intermolecular bonds, which enhanced the films’ mechanical resistance [87,88]. This result is in accordance with a previous study where 1,3-propanediol plasticized films exhibited a significant improvement in tensile strength compared to the control chitosan-exopolysaccharide films [88]. The tensile strength was decreased by using acacia gum or glycerol in the film preparation. A previous study conducted by Masti et al. has revealed that adding acacia gum to a PVA/chitosan (50/50) film enhanced the tensile strength and Young’s modulus, but further increasing the acacia gum concentration beyond a certain point reduced these properties. This study showed that incorporating acacia gum into PVA-based films can modify their mechanical properties, the effects varying based on its concentration [89]. The decrease in tensile strength of the films with the addition of glycerol can be attributed to the intercalation of glycerol molecules within the matrix network, which disrupts the intramolecular forces. This disruption can also result in a reduction in hardness values (Y5), as observed in the current study [78]. The elongation at break (Y9) values ranged between 2.45% (N10) and 176.56% (N4), as presented in Table 4. This parameter was positively influenced by the addition of glycerol and negatively influenced by xylitol (Figure 1), following the same trends and for similar reasons as observed for the deformation at target values. This impact of glycerol on elongation at break and tensile strength was similarly reported by Faust et al. [78]. With the use of a higher ratio of a co-film-forming agent, a decrease in the elongation at break values was noticed, possibly explained by the cross-linking phenomenon described above. The Young’s modulus (Y10) values ranged from 13.11 MPa (N2) to 796.25 MPa (N9), as shown in Table 4. The Young’s modulus of the films was negatively influenced by glycerol and positively influenced by xylitol (Figure 1). The Young’s modulus, also known as the elastic modulus, describes the ability of an elastic material to resist deformation to an applied stress [90]. A lower Young’s modulus indicates greater elasticity, as the material deforms more easily under an applied stress [32]. In this study, the addition of glycerol decreased the Young’s modulus, correlating with a decrease in tensile strength and an increase in the deformation of the target and elongation at break. This confirms glycerol’s good plasticizer effect and its role in reducing film stiffness, consistent with the findings from the previous studies [32,91,92]. Inversely, xylitol increased the Young’s modulus, thereby decreasing the flexibility of the films. This increase can be correlated with a decrease in the deformation of the target and elongation at break, likely due to the crystallization process of the xylitol within the film matrix, which contributed to the observed rigidity.

To summarize, concerning the previously established CQAs, an increased swelling degree was observed by association of PVA with CMC-Na or xanthan gum as co-film-forming agents in the film-dressing composition. Increased adhesive properties and hardness were also obtained by using CMC-Na, while increased deformation at the target and elongation at break were observed when glycerol was used as a plasticizer.

#### 2.7.4. Folding Endurance

This analysis showed that all formulations exhibited high folding endurance, with the films maintaining their shape and integrity after more than 300 folds, being suitable for wound dressing applications. Adequate flexibility of the film-dressings is crucial to avoid cracking or breaking and is correlated with their ability to conform to the irregular shapes of the body. This flexibility ensures that the films permit movement without causing pain or harm to the wound or surrounding tissue [93].

### 2.8. Optimization of CHE-Loaded Film-Dressing Formulation

To optimize the formulation of the film-dressings, the CQAs were subjected to a set of constraints based on the results from the DoE and on the QTPP previously established and justified. Thereby, the swelling degree, adhesive properties, deformation at target and elongation at break were maximized, while the hardness was maximized with a minimum value of 2500 g. The composition of the optimized CHE-loaded film-dressing is presented in Table 5.

After preparation of the optimized CHE-loaded film-dressing, it was analyzed through the same methods as the experiments from the DoE. The results of an analysis of the optimized CHE-loaded film-dressing, compared to the values predicted by the optimization software, are presented in Table 6.

The optimized CHE-loaded film-dressing showed properties that aligned with the initially established QTPP (Table 1). According to Table 6, the mechanical properties were found to be very good, with high hardness (Y5—2616.00 g, thus superior to the established minimum value of 2500 g), high deformation at target (Y7—29.80%) and high elongation at break (Y9—106.90%). The swelling degree also reached high values (Y2—628.28%), as did the adhesive properties (Y3—28.00 g and Y4—0.20 mJ). It was observed that the values obtained for the mechanical and adhesive properties exhibit an underprediction, which may result from prediction errors of the model and/or inadequate control of the environmental factors (temperature, humidity) during the characterization of the films. However, the results obtained demonstrated a good performance of the optimized CHE-loaded film-dressing.

The swelling degree of our optimized CHE-loaded film-dressing (Y2—628.28%, as seen in Table 6) was found to be higher than comparable formulations intended for wound care that were reported in the literature [24,49,94,95], highlighting the advantage of its use, as a high swelling degree is beneficial for absorbing wound exudate and maintaining a moist environment favorable to healing [49]. The mechanical properties were appropriate for the intended application, aligning with the previous results obtained using the same equipment in the development of other wound care film-dressings The hardness value of the optimized CHE-loaded film-dressing (Y5—2616.00 g, as shown in Table 6) was close to that reported by Savencu et al. [24], while hardness (mechanical strength) and deformation at target were found to be higher than the results obtained by Colobatiu et al. [14]. The adhesive properties (Y3—28.00 g and Y4—0.20 mJ, as observed in Table 6) proved to be comparable with the previously reported results by the same authors [14,24]. Thus, the high mechanical and adhesive properties are important advantages of the optimized CHE-loaded film-dressing for enhanced physical resistance, together with appropriate tissue adhesion to maintain the dressing in place, as previously established in the QTPP [7,31]. In addition to the targeted values for the CQAs, the remaining characteristics were also found to be desirable. It has been established that an ideal wound dressing requires high elongation at break, high tensile strength, and a low Young’s modulus to ensure durability and resistance to stress during application and handling [32]. The mechanical properties (evaluated by tensile test) of the CHE-loaded optimized film were excellent (tensile strength—Y8—5.41 MPa, elongation at break—Y9—106.90%, Young’s modulus—11.22 MPa, as presented in Table 6). The tensile strength value was within the suitable range for wound dressings, which is typically between 1 and 32 MPa, as determined by the tensile strength of human skin [47,48]. Moreover, the values for the Young’s modulus were also in the range reported for native skin (Young’s modulus ranging between 0.4 and 20 MPa) [42,43]. The literature reports that the elongation at break of wound dressings should be higher than 70% [96], a criterion that is also met by the optimized CHE-loaded film-dressing. The thickness of the CHE-loaded optimized film was also considered appropriate (Y1—0.19 mm, as observed in Table 6). According to Hoffmann et al. [35], the most common film thickness ranges between 0.012 and 0.1 mm; however, some commercially available films exceed 0.1 mm, as there are no strict regulations for standard thickness range. See also the study by Pechová et al. [97]. The thickness of the optimized CHE-loaded film-dressings was comparable with previously obtained films [14,24]. Overall, the results obtained demonstrated very good properties of the optimized CHE-loaded film-dressing to be used in wound care.

### 2.9. In Vitro CHE Release from the Optimized CHE-Loaded Film-Dressing

**Figure 2:**
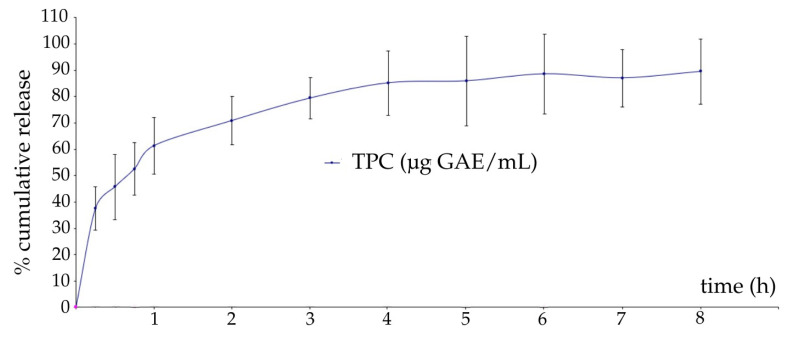
In vitro release profile of polyphenols from CHE from optimized CHE-loaded film-dressing (TPC—total polyphenolic content, GAE—gallic acid equivalents).

Figure 2 presents the in vitro release profile of polyphenols from CHE from optimized CHE-loaded film-dressing.

The release profile showed a gradual and rapid release phase within the first few hours, attributed to the diffusion of polyphenols from the film’s surface, triggering an initial burst release. This was followed by a sustained release phase, because polyphenols from the polymer matrix gradually diffused through the polymeric network during film jellification, as previously explained [98,99]. A maximum release of polyphenols of 88.00% was reached after 8 h, with 60.33% released in the first hour. The high release of the entrapped phytocompounds in the polymeric film can be correlated with the high swelling degree of the CHE-loaded optimized film-dressing. Sustained release is desirable to obtain a prolonged therapeutic effect of the herbal extracts in the wound site, ensuring effective concentrations over time [100]. The temperature during the experiment was set to 32 °C, as the temperature of the wound bed is within a range of 30.2–33.0 °C [101]. An aqueous receptor medium was chosen to mimic the watery inflammatory exudate. Ethanol was added to improve the solubility of polyphenols, ensuring that their release is not limited by saturation effects in the receptor medium, as lipophilic polyphenols being soluble in ethanol [102].

Previous research evaluated the release profile of polyphenols from chitosan-based films, using the Folin–Ciocâlteu’s method, and different receptor mediums: water, 3% acetic acid, 10%, and 20% ethanol aqueous solutions, obtaining similar trends of polyphenols release [17,103,104]. Luo et al. also found comparable polyphenol release patterns in ethanolic and aqueous receptor medium using the Folin–Ciocâlteu’s method [17]. Esmaeili et al. investigated an *Artemisia absinthium* extract-loaded soy protein isolate nanoparticle and sodium alginate film loaded with the nanocarrier. It is noteworthy that the researchers developed a new system by integrating a microfluidic device and dialysis bag as a more reliable study of drug delivery systems that is a promising method to be employed in further studies [105].

Generally, the release of polyphenols from the polymer matrix has been explained by several factors: (1) polymer properties, such as swelling degree, which determine the water diffusion in the polymeric network and the network’s relaxation, molecular weight distribution, density, and orientation, which may determine sizes and shapes of the microcavities and their distribution, and consequently the diffusion of incorporated substances; (2) polyphenols properties such as their molecular size, shape, density, polarity, and solubility; and (3) factors related to the interaction of the polymeric matrix with the polyphenols, such as the plasticizing or anti-plasticizing effects of the polyphenols on the polymeric network [17,103]. According to the fitting of the release data with the Korsmeyer–Peppas kinetic model, the release index (n) was calculated to be 0.35. Since n is less than 0.45, this indicates that the drug release follows a Fickian diffusion mechanism, meaning that the active compound diffuses through the matrix in a concentration-dependent manner, as described by Fick’s laws of diffusion [17,106].

## 3. Conclusions

This study effectively highlighted the potential of using a QbD approach to design and optimize film-dressings loaded with herbal extracts. By systematically analyzing the impact of key excipients, such as co-film-forming agents (CMC-Na, xanthan gum, acacia gum) and plasticizers (glycerol, xylitol, 1,3-propanediol) on the properties of the film-dressings, important influences have been observed. Subsequently, an increased swelling degree was observed by association of the main film-forming agent, namely PVA, with CMC-Na or xanthan gum. Increased adhesive properties and hardness were obtained by using CMC-Na, while increased deformation at target and elongation at break were observed by adding glycerol. The optimized formulation containing 10% PVA, 0.25% xanthan gum, 10% glycerol, and 20% complex herbal extract achieved excellent quality attributes, including both high mechanical strength and high flexibility (hardness of 2616.00 g, rigidity at 5 mm of 593 g, deformation at target of 29.80%, tensile strength of 5.41 MPa, elongation at break of 106.90%, and Young’s modulus of 11.22 MPa), good adhesive properties (adhesive force of 28.00 g and adhesiveness of 0.20 mJ), high swelling degree (628.28%), and an appropriate thickness of 0.19 mm. In vitro release studies revealed a sustained release of polyphenols over 8 h, showing the optimized film-dressing’s ability to provide prolonged therapeutic effects. These properties of the optimized film dressing are essential for ensuring adequate physical resistance, maintaining a moist wound environment, enhancing patient comfort during treatment, and effectively supporting the wound healing process. The findings of this study can serve as a valuable reference for developing film-dressings loaded with herbal extracts using the QbD approach, offering a promising solution for advanced wound care applications. Future studies could evaluate the optimized film-dressing’s stability and efficacy through in vitro and in vivo studies.

## 4. Materials and Methods

### 4.1. Chemicals and Reagents

Acacia gum, xanthan gum, and xylitol were purchased from Ellemental, Oradea, Romania. Glycerol, ethanol, and sodium carbonate were acquired from International Laboratory, Cluj-Napoca, Romania. Other substances used included: 1,3-propanediol (Ellemental, Oradea, Romania), Folin–Ciocâlteu reagent (ChemPUR, Piekary Śląskie, Poland), gallic acid (Merck, Darmstadt, Germany), polyvinyl alcohol (PVA) MW 195.000 Da, polymerization degree 4300, (Kuraray Poval^®^56–98, Tokyo, Japan), and sodium carboxymethylcellulose (CMC-Na) medium viscosity (FLUKA, Biochemika, Buchs, Switzerland).

### 4.2. The QbD Approach

#### 4.2.1. Establishment of the QTPP and CQAs of the CHE-Loaded Film-Dressings

The QTPP defines the desired properties and ensures the final product meets the technological and therapeutic needs. The CQAs for film-dressings should be specific physical and biological properties that must be controlled during the optimization step inclusively by setting the acceptance criteria, to ensure the product meets its QTPP [25,26].

#### 4.2.2. Risk Analysis

To identify and assess potential risks in the development of the film dressings, a structured risk analysis was performed using both the Ishikawa diagram (Fishbone diagram) and the failure mode and effects analysis (FMEA) (Figure S1 and Table S1). The Ishikawa diagram was used to systematically visualize and categorize the possible sources of variation during the development process. Following this, FMEA was employed to evaluate the severity, occurrence, and detectability of identified risks, assigning risk priority numbers (RPNs) to prioritize those requiring mitigation strategies [25,26].

#### 4.2.3. Development of the DoE

To study the influences of the formulation factors on the responses represented by the quality parameters of the films, a D-optimal experimental plan with N = 18 experiments (Table 7) was developed using Modde 13 software (Sartorius Stedim, Göttingen, Germany).

### 4.3. Preparation of the CHE-Loaded Film-Dressings

The solvent casting method was used to prepare the film-dressings, as previously described [14,24]. The solvent casting method was chosen to prepare the film-dressings, due to its simplicity and the cost-effectiveness of the process, leading to the obtention of films with good physical properties and excellent uniformity of thickness if the drying surface is flat [9]. The film-forming agent PVA was used in a 5% ratio for all experiments, previously dissolved in water to obtain a 10% stock solution. The complex herbal extract (CHE) consisting of hydroethanolic extracts from *Sambucus nigra* flowers, *Epilobium hirsutum* aerial parts, and *Lythrum salicaria* aerial parts was optimized in a previous study for inclusion in wound care applications [20]. The CHE was used in a 20% ratio for all film-dressings. The formulations from the experimental plan were prepared by mixing the excipients (PVA with xanthan gum, acacia gum, or CMC-Na with glycerol, xylitol, or 1,3-propanediol) with the CHE and water to obtain a homogenous gel. The gel was poured into 10 cm diameter polyethylene Petri dishes. The film dressings were dried at room temperature (20 ± 2 °C, 50–60% relative humidity) for 48 h. Once dried, the films were peeled off and analyzed.

### 4.4. Characterization of the CHE-Loaded Film-Dressings

These characterization methods were used for all the formulation from the DoE. All the analyses were performed in triplicate and the mean ± standard deviation was reported.

#### 4.4.1. Organoleptic Evaluation

After the preparation of the film-dressings, they were visually inspected for appearance and homogeneity and examined for handling ease.

#### 4.4.2. Uniformity of Mass

The samples were cut into 2 cm × 2 cm square shapes and weighed individually. The average mass of 10 samples was calculated [24,34].

#### 4.4.3. Film Thickness

The thickness of the films was measured using a digital electronic micrometer (Mitutoyo, Tokyo, Japan) at 8 different locations [24,107].

#### 4.4.4. Swelling Degree

The samples were cut into 2 cm × 2 cm square shapes and were weighed to determine the mass before swelling (m_i_—mass of initial film, dry). Each sample was immersed for 2 h at 22 ± 2 °C in a Petri dish with a volume of 32 cm^3^ of distilled water, following the recommendations of ISO 175:2010 (8 mL of medium/cm^2^ sample) [108,109]. The excess water was removed by absorbing with a tissue, and the samples were weighed to determine the mass after swelling (m_f_—mass of the swelled film). The swelling degree was calculated according to the following formula [9,14,110]:

(2) $$Swelling\;degree\;=\;\frac{m_{f}-m_{i}}{m_{i}}\;\times 100$$

#### 4.4.5. Mechanical Properties—Puncture Test

The mechanical properties of the film-dressings were evaluated using the Texture Analyzer CT3 (Brookfield Engineering Laboratories, Middleborough, MA, USA), coupled with the TA-FSF fixture and a cylindrical probe with rounded bottom with ⌀0.8 cm and 6 cm length. The rupture test was made at the test speed of 0.2 mm/s and trigger load of 10.0 g. The determined parameters were hardness (g), rigidity at 5 mm (g), and deformation at target (mm). Hardness was measured as the force required to cause a certain deformation, in our case a rupture of the film by the probe. Deformation at target was measured as the total downward distance that the attachment causes on the film once the trigger load is reached [14,111].

#### 4.4.6. Mechanical Properties—Tensile Test

The elastic properties of the film-dressings were evaluated using the same Texture Analyzer CT3, equipped with TA-DGF fixture. The 4 cm × 2 cm pre-cut samples were fixed between the grips of the fixture and further analyzed by using the following settings: Test type: Tension; Target Type: Distance; Target load: 90 mm; Test speed: 0.5 mm/s. The peak load (g) and deformation at peak load (mm) were registered using Texture Pro CT V1.9 software [111], and the tensile strength (MPa), elongation at break (%), and the Young’s modulus (MPa) were calculated according to the following formulas:

(3) $$Tensile\;strength\;=\;\frac{F_{max}}{A}$$

(4) $$Elongation\;at\;break\;=\;\frac{L_{max}}{L_{i}}\times 100\;$$

(5) $$Young’s\;modulus\;=\frac{L_{i}}{A}\;\times S$$

where F_max_ is the peak load in N, L_max_ is the maximum deformation before breakage (deformation at peak load in mm), L_i_ is the initial length (40 mm), A is the cross-sectional area of the film in mm^2^, and S is the slope of the linear part of the force vs. deformation curve [112].

#### 4.4.7. Adhesive Properties

The adhesive properties of the film-dressings were evaluated using the same Texture Analyzer CT3, equipped with a TA-AACC36 probe. The film was cut and attached to the bottom of the cylinder probe with double-sided adhesive tape). Prior to the analysis, 20 g of 6.67% (*w*/*v*) gelatin were poured into a Petri dish (⌀90 mm) and maintained in the refrigerator overnight to form a gel. An amount of 500 µL of distilled water was uniformly applied to the tested area and left to moisten for 30 s, to simulate a wound surface with aqueous exudate [113]. Subsequently, the excess water was absorbed with paper tissue. Then, the compression test was made with the target type Stop & Load, target value of 100 g, hold time of 60 s, trigger load of 5 g, and test speed of 0.10 mm/s. The adhesiveness and the adhesive force were measured as the energy and force required to separate the substrate from the sample [14,24,111].

#### 4.4.8. Folding Endurance

The folding endurance was determined by bending and overlapping the 4 cm × 2 cm pre-cut samples at an angle of 180° until the film either broke or developed visible cracks. The folding endurance was expressed as the number of folds required to reach the failure point. A film is considered tear-resistant if it withstands a minimum of 300 folds without breaking or cracking. This analysis provides information on the fragility of the film and its resistance during packaging and handling [9,24,39].

### 4.5. Optimization of CHE-Loaded Film-Dressing Formulation

The optimization of the film-dressing formulation was made using Modde 13 software (Sartorius Stedim, Göttingen, Germany) by maximizing the swelling degree, adhesiveness and adhesive force, deformation at target, elongation at break, and hardness by setting a minimum value of 2500 g. The optimized film-dressing was further analyzed in terms of the parameters evaluated in the experimental plan to compare the results with the ones predicted by the optimization program.

### 4.6. In Vitro CHE Release from the Optimized CHE-Loaded Film-Dressing

The in vitro release of the phytocomponents from the film-dressing was studied using an automated Phoenix diffusion cell system (Teledyne Hanson, Chatsworth, CA, USA). The receptor medium consisting of a 25:75 (*v*/*v*) an ethanol/water solution was maintained under magnetic stirring (400 rpm) at 32 ± 0.5 °C. Circular samples were randomly cut from the CHE-loaded film-dressing and carefully placed on the receptor compartment, ensuring proper contact with it through the 9 mm diameter opening of the cell cap. To prevent evaporation during the study a glass cover was used. The released amount of the phytocompounds from the CHE-loaded film-dressing was determined at specific intervals of time (15, 30, 45 min, 1 h, and then hourly for 8 h) by collecting 500 µL of medium from the receptor compartment using the autosampler. The collected volume was immediately replaced by the same volume of fresh medium. The measurement was performed in triplicate and the cumulative release (%) was calculated and graphically represented against time [24,102].

The total amount of the film with a 9 mm diameter was quantified after subjecting the sample to sonication in an ultrasonic bath for 2 h in 10 mL of receptor medium. Following sonication, the mixture was centrifuged for 30 min at 10,000 rpm to separate the components for further analysis.

The released amount of phytocomponents was determined by quantifying the total polyphenolic content according to the method previously used to optimize the herbal extracts [20]. The total amount of polyphenols from samples with 9 mm diameter was quantified after sonication of the film in an ultrasonic bath for 2 h in 10 mL of receptor medium. Following sonication, the mixture was centrifuged for 30 min at 10,000 rpm [14]. Then, 60 µL of sample was mixed with 270 µL of the Folin–Ciocâlteu reagent (freshly diluted 1:10 with distilled water) and 270 µL of Na_2_CO_3_ solution 6% *w*/*v*) [114]. After incubation at room temperature for 30 min in the dark, the absorbances were recorded at 760 nm using receptor medium as blank solution in the UV-VIS spectrophotometer (Specord^®^ 200 Plus, Analytik Jena, Germany). The calibration curve was determined using gallic acid dissolved in receptor medium in a concentration range of 10–50 µg/mL (R^2^—0.9959). The results were expressed as micrograms of gallic acid equivalents (GAE) per milliliter (µg GAE/mL). Previously, preliminary analyses were made to determine the most suitable medium for the study [115]. The tested media included water, phosphate buffer (pH 6), acetate buffer (pH 6) and 75% ethanol/25% water mixture.

To further understand the kinetic mechanism of polyphenol release from the optimized CHE-loaded film-dressing, the release data were fitted to Korsmeyer–Peppas kinetic model and the release index (n) was used to explain the diffusion mechanism [17,104].

### 4.7. Statistical Analysis

All samples were analyzed in triplicate and the results were presented as a mean ± standard deviation (SD). The data obtained from the DoE were statistically analyzed by performing ANOVA analysis in Modde 13 software. The results were considered statistically significant if *p* values were lower than 0.05.

### 4.8. Study Limitations

In the present study, our focus was the formulation optimization using a quality by design approach and a design of experiments method, which involved analyzing multiple formulation variables affecting various film characteristics. The limitation of this study is that the optimized CHE-loaded film-dressing was not assessed for microbiological or cytotoxicity properties, as we focused on the characterization of the film-dressings and not on the biological evaluation. In the previous study regarding the optimization and evaluation of the biological properties of the three herbal extracts, we conducted microbiological and cytotoxicity evaluations, obtaining promising results. Additionally, anti-inflammatory and wound healing effects were demonstrated, which guided us to include these extracts in film dressings for wound care [20]. However, given the importance of these evaluations on the final pharmaceutical form too, we acknowledge their relevance for future investigations.

## Figures and Tables

**Table 2:** Statistical parameters for ANOVA test and quality of fit.

Response	R^2^	Q^2^	*p*-Value	Lack of Fit	Model Validity	Reproducibility
Y1	0.778	0.732	0.000	0.107	0.440	0.964
Y2	0.710	0.531	0.006	0.067	0.322	0.966
Y3	0.549	0.397	0.003	0.070	0.336	0.957
Y4	0.712	0.482	0.002	0.323	0.717	0.832
Y5	0.579	0.313	0.017	0.427	0.787	0.655
Y6	0.703	0.419	0.006	0.028	0.109	0.985
Y7	0.781	0.661	0.004	0.440	0.794	0.775
Y8	0.885	0.697	0.000	0.086	0.385	0.980
Y9	0.525	0.327	0.013	0.248	0.651	0.812
Y10	0.785	0.753	0.000	0.194	0.589	0.940

Y1—film thickness (mm), Y2—swelling degree (%), Y3—adhesive force (g), Y4—adhesiveness (mJ), Y5—hardness (g), Y6—rigidity at 5 mm (g), Y7—deformation at target (%), Y8—tensile strength (MPa), Y9—elongation at break (%), Y10—Young’s modulus (MPa).

**Table 3:** Uniformity of mass of the CHE-loaded film-dressings.

Exp.	Average Mass (g)	Exp.	Average Mass (g)
N1	0.0540 ± 0.0070	N10	0.0746 ± 0.0191
N2	0.1456 ± 0.0327	N11	0.0693 ± 0.0155
N3	0.0720 ± 0.0173	N12	0.0543 ± 0.0095
N4	0.1183 ± 0.0143	N13	0.9039 ± 0.0097
N5	0.1286 ± 0.0372	N14	0.0423 ± 0.0025
N6	0.0770 ± 0.0108	N15	0.1263 ± 0.0120
N7	0.0646 ± 0.0108	N16	0.1120 ± 0.0401
N8	0.0906 ± 0.0253	N17	0.1193 ± 0.0217
N9	0.0792 ± 0.0233	N18	0.0983 ± 0.0140

**Table 4:** DoE and matrix of the results.

Exp.	X1	X2	X3	X4	Y1 (mm)	Y2 (%)	Y3 (g)	Y4 (mJ)	Y5 (g)	Y6 (g)	Y7 (mm)	Y8 (MPa)	Y9 (%)	Y10 (MPa)
N1	AG	G	−1	−1	0.13 ± 0.01	907.86 ± 66.60	62.20 ± 5.00	0.27 ± 0.03	4219.00 ± 315.00	1111.00 ± 278.00	30.49 ± 9.25	8.24 ± 2.26	70.44 ± 11.01	76.20 ± 31.84
N2	AG	G	1	1	0.20 ± 0.03	446.61 ± 17.68	36.00 ± 1.70	0.18 ± 0.01	2485.00 ± 115.00	669.00 ± 97.00	29.89 ± 3.38	4.58 ± 0.33	115.12 ± 36.39	13.11 ± 4.01
N3	XG	G	1	−1	0.19 ± 0.05	2932.04 ± 1444.75	62.50 ± 22.40	0.30 ± 0.05	6946.00 ± 872.00	1823.00 ± 471.00	19.19 ± 4.66	13.84 ± 2.46	93.30 ± 25.62	35.59 ± 10.46
N4	XG	G	−1	1	0.17 ± 0.04	555.26 ± 41.74	40.70 ± 7.80	0.25 ± 0.03	4273.00 ± 613.00	788.00 ± 116.00	36.70 ± 6.18	9.41 ± 1.08	176.56 ± 20.32	24.22 ± 4.50
N5	CMC-Na	G	−1	1	0.20 ± 0.07	1126.05 ± 81.61	84.80 ± 3.70	0.25 ± 0.04	3523.00 ± 291.00	697.00 ± 60.00	35.99 ± 7.19	6.88 ± 0.66	169.92 ± 20.62	16.09 ± 5.40
N6	CMC-Na	G	1	−1	0.12 ± 0.04	1653.08 ± 82.24	52.10 ± 22.30	0.32 ± 0.11	8019.00 ± 1533.00	2890.00 ± 819.00	11.21 ± 2.52	22.71 ± 1.83	11.26 ± 3.45	196.19 ± 93.21
N7	AG	X	−1	−1	0.10 ± 0.04	970.69 ± 74.64	13.00 ± 2.60	0.15 ± 0.02	3948.00 ± 257.00	1882.00 ± 496.00	13.66 ± 2.33	23.75 ± 1.56	66.77 ± 17.57	361.14 ± 73.84
N8	AG	X	1	1	0.11 ± 0.05	889.29 ± 35.17	25.30 ± 6.70	0.20 ± 0.06	5621.00 ± 1892.00	2750.00 ± 574.00	12.53 ± 2.98	16.25 ± 2.85	23.27 ± 18.95	352.76 ± 25.54
N9	XG	X	1	−1	0.12 ± 0.02	3560.62 ± 1095.17	69.00 ± 8.50	0.32 ± 0.01	6771.00 ± 964.00	3203.00 ± 450.00	9.87 ± 0.46	36.30 ± 11.43	4.73 ± 4.62	796.25 ± 260.43
N10	XG	X	−1	1	0.13 ± 0.05	2398.01 ± 498.766	37.80 ± 7.70	0.18 ± 0.10	6650.00 ± 926.00	5051.00 ± 374.00	7.80 ± 1.77	33.54 ± 4.93	2.45 ± 1.26	704.89 ± 36.74
N11	CMC-Na	X	−1	−1	0.12 ± 0.03	1721.66 ± 232.28	52.20 ± 13.70	0.34 ± 0.07	8543.00 ± 1476.00	3096.00 ± 494.00	12.01 ± 1.78	34.18 ± 6.81	48.99 ± 7.58	412.93 ± 135.39
N12	AG	P	1	−1	0.10 ± 0.04	951.20 ± 126.18	22.80 ± 3.80	0.16 ± 0.02	3985.00 ± 376.00	1926.00 ± 293.00	12.93 ± 1.35	24.72 ± 1.78	89.36 ± 24.57	183.27 ± 21.400
N13	AG	P	−1	1	0.15 ± 0.03	897.19 ± 111.37	31.50 ± 11.00	0.18 ± 0.04	5856.00 ± 1267.00	1456.00 ± 208.00	23.00 ± 6.26	31.49 ± 14.66	143.90 ± 60.70	246.19 ± 35.27
N14	XG	P	−1	−1	0.10 ± 0.03	1907.52 ± 318.88	53.80 ± 16.30	0.25 ± 0.07	7166.00 ± 1054.00	1710.00 ± 334.00	22.32 ± 3.79	34.4 ± 7.24	130.85 ± 64.30	108.63 ± 8.91
N15	CMC-Na	P	0	1	0.11 ± 0.05	1928.64 ± 65.72	72.80 ± 13.80	0.29 ± 0.06	8556.00 ± 984.00	2456.00 ± 201.00	16.11 ± 1.83	29.71 ± 3.91	51.86 ± 17.95	213.88 ± 37.67
N16	CMC-Na	G	0	0	0.19 ± 0.04	1532.19 ± 37.66	64.70 ± 17.20	0.31 ± 0.01	4547.00 ± 1687.00	896.00 ± 459.00	23.21 ± 7.15	13.11 ± 0.88	142.36 ± 25.24	33.41 ± 1.05
N17	CMC-Na	G	0	0	0.18 ± 0.05	1608.00 ± 114.15	60.20 ± 10.80	0.30 ± 0.02	6658.00 ± 618.00	1027.00 ± 296.00	31.93 ± 5.69	10.76 ± 3.97	98.03 ± 38.41	22.81 ± 11.28
N18	CMC-Na	G	0	0	0.19 ± 0.03	1327.00 ± 93.27	56.7 ± 10.30	0.26 ± 0.04	5609.00 ± 1260.00	996.00 ± 107.00	26.13 ± 3.36	13.58 ± 1.87	124.05 ± 26.27	43.57 ± 11.5

AG—acacia gum, XG—xanthan gum, CMC-Na—sodium carboxymethylcellulose, G—glycerol, X—xylitol, P—1,3-propanediol, X1—type of co-film-forming agent, X2—type of plasticizer, X3—concentration of co-film-forming agent, X4—concentration of plasticizer, Y1—film thickness (mm), Y2—swelling degree (%), Y3—adhesive force (g), Y4—adhesiveness (mJ), Y5—hardness (g), Y6—rigidity at 5 mm (g), Y7—deformation at target (mm), Y8—tensile strength (MPa), Y9—elongation at break (%), Y10—Young’s modulus (MPa).

**Table 5:** The composition of the optimized CHE-loaded film-dressing and the results of its analysis.

Composition of Optimized CHE-Loaded Film-Dressing	
Ingredient	Concentration (%)
Polyvinyl alcohol	5.00
Xanthan gum	0.25
Glycerol	10.00
CHE	20.00
Distilled water	Qs ad 100

**Table 6:** Results of analysis of the optimized CHE-loaded film-dressing.

	Y1 (mm)	Y2 (%)	Y3 (g)	Y4 (mJ)	Y5 (g)	Y6 (g)	Y7 (%)	Y8 (MPa)	Y9 (%)	Y10 (MPa)
Predicted values	0.19	1223.33	52.76	0.22	5293.70	1104.32	32.89	12.86	135.17	35.52
Experimental values	0.19 ±0.04	627.28 ±85.23	28.00 ±6.00	0.20 ±0.00	2616.00 ±409.00	593.00 ±100.00	29.80 ±4.75	5.41 ±0.93	106.90 ±9.76	11.22 ±3.90
Residual	0	−596.05	−24.76	−0.02	−2677.7	−511.32	−3.09	−7.45	−28.27	−24.3

Y1—film thickness (mm), Y2—swelling degree (%), Y3—adhesive force (g), Y4—adhesiveness (mJ), Y5—hardness (g), Y6—rigidity at 5 mm (g), Y7—deformation at target (%), Y8—tensile strength (MPa), Y9—elongation at break (%), Y10—Young’s modulus (MPa).

**Table 7:** DoE of the CHE-loaded film-dressings.

Independent Variables (Formulation Factors)						Dependent Variables (Responses)
Qualitative Variables		Quantitative Variables				
X1—Type of co-film-forming agent	XG	X3—Concentration of co-film-forming agent	Variation levels (%)			Y1—Film thickness (mm), Y2—Swelling degree (%), Y3—Adhesive force (g), Y4—Adhesiveness (mJ), Y5—Hardness (g), Y6—Rigidity la 5 mm (g), Y7—Deformation at target (mm), Y8—Tensile strength (MPa), Y9—Elongation at break (%), Y10—Young’s modulus (MPa).
			−1	0	1	
			0.25	0.375	0.5	
	AG		0.5	0.75	1.5	
	CMC-Na		1	1.5	2	
X2—Type of plasticizer	G	X4—Concentration of plasticizer	3	6.5	10	
	X		3	4	5	
	P		3	4	5	

XG—xanthan gum, AG—acacia gum, CMC-Na—sodium carboxymethylcellulose, G—glycerol, X—xylitol, P—1,3-propanediol.

## Data Availability

The raw data supporting the conclusions of this article will be made available by the authors upon request.

## Supplementary Materials

The following supporting information can be downloaded at: https://www.mdpi.com/article/10.3390/gels11050322/s1, Figure S1. Ishikawa diagram of CHE-loaded film-dressings; Table S1. FMEA Risk assessment for CHE-loaded film-dressings; Figure S2. Replicate plots; Figure S3. Contour 4D plots; Table S2. Revised quantitative factor effects and the associated *p*-values; Table S3. Descriptive statistics of the DoE; Table S4. Statistics of the model.

## Abbreviations

The following abbreviations are used in this manuscript:

CHE	Complex Herbal Extract
QbD	Quality by Design
DoE	Design of Experiments
WVTR	Water Vapor Transmission Rate
WCA	Water Contact Angle
QTPP	Quality Target Profile Product
CQAs	Critical Quality Attributes
