# Supplementary material for: Optimization of Film-Dressings Containing Herbal Extracts for Wound Care—A Quality by Design Approach
Source: Gels. 2025 Apr 25;11(5):322. doi: 10.3390/gels11050322 (PMC12111743; doi:10.3390/gels11050322)
Supplement: Supplementary file 1 [file gels-11-00322-s001.zip › gels-3555252-supplementary.pdf]

Supplementary materials

Optimization of Film-Dressings containing Herbal Extracts for Wound Care - a Quality by Design Approach

Diana Antonia Safta[1], Cătălina Bogdan*[1,2], Sonia Iurian[3], Mirela L. Moldovan[1]

[1] Department of Dermopharmacy and Cosmetics, Faculty of Pharmacy, "Iuliu Haţieganu" University of Medicine and Pharmacy, 12 I. Creangă St. 400010 Cluj-Napoca, Romania
2 Department 2, Faculty of Nursing and Health Sciences, "Iuliu Haţieganu" University of Medicine and Pharmacy, 4 L. Pasteur St. 400349 Cluj-Napoca, Romania
3 Department of Pharmaceutical Technology and Biopharmacy, Faculty of Pharmacy, "Iuliu Haţieganu" University of Medicine and Pharmacy, 41 V. Babes, St. 400012, Cluj-Napoca, Romania

* Correspondence: catalina.bogdan@umfcluj.ro

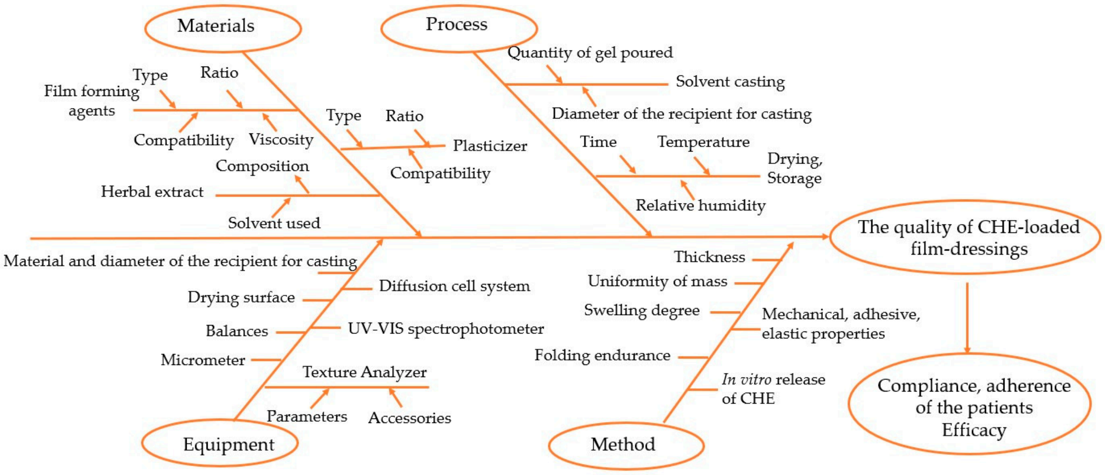

Figure S1. Ishikawa diagram of CHE-loaded film-dressings.

Table S1. FMEA Risk assessment for CHE-loaded film-dressings.

Variation factors		Potential failure mode	Potential failure effects	Control method/Correction action	O	S	D	RPN
				**CMAs**				
**Film forming agents**	Type	Interactions between chemical groups in structure of ingredients resulting in problems with film formation and film properties	Non-adequate physical properties	Establishment of certain film forming agents to be studied by DoE, based on preliminary formulations	5	5	3	75
	Ratio	Improper concentration (too high - too high viscosity of gel; too little - difficulties in film formation)	Non-adequate physical properties	Establishment of an appropriate interval of concentrations to be studied by DoE, based on preliminary formulations	5	5	4	100
	Compatibility	Incompatibility with other ingredients	Non-homogeneity, instability	Checking of the ingredients compatibility in specialty literature and based on preliminary formulations	2	5	1	10
	Viscosity	Too high viscosity - formation of air bubbles during the preparation, uneven gel pouring, thickness inconsistency	Non-homogeneity, weak points in mechanical strength	Adjustments of polymer types and concentration on preliminary formulations	4	4	1	16
Plasticizer	Type	Interactions between chemical groups in structure of ingredients resulting in problems with film formation and film properties	Non-adequate physical properties	Establishment of certain plasticizers to be studied by DoE, based on preliminary formulations	5	5	3	75
	Ratio	Improper concentration (too high: sticky surface, uncomfortable for the user, leaching out of plasticizer; too little: brittle and non-flexible films)	Non-adequate physical properties	Establishment of an appropriate interval of concentrations to be studied by DoE, based on preliminary formulations	5	5	4	100
	Compatibility	Incompatibility with other ingredients, Interactions between chemical groups in structure of ingredients	Non-homogeneity, instability	Checking of the ingredients compatibility in specialty literature and based on preliminary formulations	2	5	1	10
Herbal extract	Composition	Non-efficiency of the extracts	Non-efficiency of the film-dressing with extracts	The extracts were thoroughly characterized in a prior study concerning their wound healing properties	1	5	2	10
	Ratio	Improper concentration	Non-adequate physical properties	Establishment of an appropriate high concentration based on preliminary formulations that was kept constant	2	4	1	8
	Compatibility	Incompatibility with other ingredients, alteration of phytocompounds	Non-homogeneity, instability	Checking of the ingredients compatibility in specialty literature and based on preliminary formulations	1	5	1	5
				**CPPs**				

Preparation	Homogeneity of gel	Incomplete mixing due to human error resulting in non-uniform gel and further film, affecting mechanical properties and release profile.	Non-homogeneity, non-adequate physical properties, non-adequate release profile	Use a mechanical stirrer; perform visual or microscopic inspection to ensure uniformity	5	5	1	25
	Quantity of poured gel	Excess gel creates thicker films, while insufficient gel causes thinner films, leading to non-uniform results.	Variability in film thickness	Establishment of an appropriate quantity of poured gel based on preliminary formulations that was kept constant	2	5	1	10
	Diameter of the recipient for casting	Variations in the diameter of the recipient where the gel is poured affect the surface area	Variability in film thickness	Use Petri dishes of standardized dimensions for all formulations	1	5	1	5
Drying, storage	Time	Insufficient drying time leads to incomplete drying; excessive time can degrade sensitive components.	Variability in film formation	All the films were dried under identical conditions within the shortest feasible time	3	5	2	30
	Temperature	High temperatures can cause degradation, while low temperatures lead to incomplete drying.	Variability in film formation	All the films were dried under identical conditions within the shortest feasible time in a controlled room to prevent excessive variations	2	5	1	10
	Relative humidity	High humidity causes stickiness or incomplete drying; low humidity leads to brittle films.	Variability in film formation	All the films were dried under identical conditions within the shortest feasible time in a controlled room to prevent excessive variations	3	5	1	15
**Equipment**								
Material and diameter of the recipient for casting		Reactive material may interact with the gel or herbal extracts, Variations in recipient size affect the surface area	Variability in film formation and film thickness	Usage of Petri dish of inert materials and of standardized dimensions for all formulations	5	5	1	25
Drying surface		A surface that is not perfectly flat leads to different film spreading and varying thickness	Variability in film thickness	Assuring a flat drying surface for all the formulations	5	5	1	25
Texture Analyzer		Improper calibration, parameters setting and accessories choosing	Inaccurate mechanical, adhesive and elastic properties measurements	Usage of appropriate parameters and accessories according to specific literature and producer's recommendations, previously established through analysis of preliminary formulations	5	5	1	25

Analytical balance	Inaccurate weighing leads to incorrect formulation ratios	Variability in film formation, film thickness, non-adequate physical properties, non-adequate release profile	Frequently calibration and usage of appropriate weighing techniques	5	5	1	25
Micrometer	Inaccurate thickness measurements	Variability in film thickness	Calibration of the micrometer and multiple readings across the film for accuracy	5	5	1	25
pHmeter	Inaccurate pH readings	Variability of the results, safety of the films	Calibration with standard buffers before usage	5	5	1	25
Diffusion cell system	Inaccurate diffusion rate measurements (non-adequate temperature, time, stirring conditions, receptor medium)	Variability of the results, efficiency of the films	Usage of appropriate parameters and receptor medium according to specific literature and producer's recommendations, previously established through analysis of preliminary formulations	5	5	1	25
			**Methods**				
Thickness	Non-reliable results	Variability of the results, efficiency of the films	Calibration and monitorization of the equipment and the method, verifying the fitting of the results by DoE	5	5	2	50
Uniformity of mass	Non-reliable results	Variability of the results, efficiency of the films	Calibration and monitorization of the equipment and the method	5	5	2	50
Swelling degree	Non-reliable results	Variability of the results, compliance and adherence of the patients, efficiency of the films	Calibration and monitorization of the method, verifying the fitting of the results by DoE	5	5	2	50
Folding endurance	Non-reliable results	Variability of the results, compliance and adherence of the patients, efficiency of the films	Calibration and monitorization of the method	5	5	2	50
Mechanical, adhesive, elastic properties	Non-reliable results	Variability of the results, compliance and adherence of the patients, efficiency of the films	Calibration and monitorization of the equipment and the method, verifying the fitting of the results by DoE	5	5	2	50
In vitro release of CHE	Non-reliable results	Variability of the results, efficiency of the films	Calibration and monitorization of the method, selection of adequate receptor medium and quantification method for the released herbal extract	5	5	2	50

CMAs – critical materials attributes, CPPs – critical process parameters, O – occurrence, S – severity, D- detectability, RPN - risk priority number

Table S2. Revised quantitative factor effects and the associated p-values.

Response	a	X1 (AG)	X1 (XG)	X1 (CMCNa)	X2 (G)	X2 (X)	X2 (P)	X3	X4
Y1 - Film thickness (mm)	0.15	-	-	-	0.03 (0.00)	- 0.02 (0.02)	- 0.02 (0.02)	-	0.01 (0.01)
Y2 - Swelling degree (%)	3.12	- 0.19 (0.00)	0.11 (0.02)	0.08 (0.09)	-0.09 (0.04)	0.06 (0.11)	0.03 (0.10)	-	-0.08 (0.08)
Y3 – Adhesive force (g)	50.35	-15.40 (0.00)	2.46 (0.54)	12.94 (0.00)	-	-	-	-	-
Y4 – Adhesiveness (mJ)	0.25	-0.05 (0.00)	0.01 (0.37)	0.04 (0.00)	-	-	-	0.01 (0.17)	-0.02 (0.02)
Y5 – Hardness (g)	5743.06	-1373.67 (0.00)	475.54 (0.23)	898.13 (0.04)	-1157.77 (0.01)	510.18 (0.25)	647.59 (0.15)	-	-
Y6 - Rigidity at 5 mm (g)	3.21	-0.11 (0.04)	0.07 (0.15)	0.03 (0.51)	-0.23 (0.00)	0.16 (0.01)	0.07 (0.23)	0.06 (0.16)	-
Y7 - Deformation at target (%)	20.83	2.13 (0.20)	-1.08 (0.50)	-1.05 (0.53)	7.86 (0.00)	-5.51 (0.01)	-2.36 (0.20)	-3.52 (0.02)	2.66 (0.07)
Y8 - Tensile strength (MPa)	20.41	-4.50 (0.01)	2.92 (0.04)	1.58 (0.27)	-10.63 (0.00)	5.05 (0.01)	5.58 (0.00)	-0.59 (0.61)	-2.25 (0.07)
Y9 - Elongation at break (%)	82.45	-	-	-	36.65 (0.00)	-30.95 (0.03)	-5.69 (0.66)	-20.59 (0.06)	-
Y10 - Young's modulus (MPa)	2.02	-	-	-	-0.53 (0.00)	0.46 (0.00)	0.07 (0.43)	-	-

AG – Accacia gum, XG – Xanthan gum, CMCNa - Sodium carboxymethylcellulose, G – Glycerol, X – Xylitol, P – 1,3 –Propanediol, X1 – Type of co-film forming agent, X2 - Type of plasticizer, X3- Concentration of co-film forming agent, X4- Concentration of plasticizer

Table S3. Descriptive statistics of the DoE.

Worksheet statistics	Y1 (mm)	Y2 (%)	Y3 (g)	Y4 (mJ)	Y5 (g)	Y6 (g)	Y7 (mm)	Y8 (MPa)	Y9 (%)	Y10 (MPa)
Min	0.10	2.65	13.00	0.15	2485.00	2.82	7.80	4.58	2.45	1.12

Max	0.21	3.55	84.80	0.36	8556.00	3.70	36.70	36.3	176.56	2.90
Mean	0.15	3.12	50.35	0.25	5743.06	3.21	20.83	20.41	82.45	2.02
Q (25%)	0.12	2.96	36.00	0.18	4219.00	2.99	12.53	10.76	45.51	1.52
Q (75%)	0.19	3.28	62.50	0.30	6946.00	3.44	29.89	31.49	120.05	2.55
Median	0.13	3.15	55.25	0.25	5738.50	3.24	20.75	19.48	79.67	2.15
Std. dev.	0.09	0.24	19.37	0.06	1797.66	0.26	9.36	10.87	52.99	0.58
Min/Max	0.49	0.75	0.1	0.4	0.29	0.76	0.21	0.13	0.014	0.38
Std.dev./Mean	0.25	0.07	0.38	0.25	0.31	0.08	0.45	0.53	0.64	0.28
Skewness	0.31	-0.16	-0.31	-0.13	-0.02	0.10	0.32	0.12	0.16	-0.10
Skewness test	0.58	-0.29	-0.57	-0.24	-0.04	0.19	0.60	0.23	0.30	-0.19
Kurtosis	-1.61	-0.18	-0.63	-1.15	-0.97	-0.97	-1.24	-1.58	-0.76	-1.38

Y1 - Film thickness (mm), Y2 - Swelling degree (%), Y3 – Adhesive force (g), Y4 – Adhesiveness (mJ) Y5 – Hardness (g), Y6 - Rigidity at 5 mm (g), Y7 - Deformation at target (mm), Y8 - Tensile strength (MPa), Y9 - Elongation at break (%), Y10 - Young's modulus (MPa).

Table S4. Statistics of the model.

**Model statistics**	**Y1 (mm)**	**Y2 (%)**	**Y3 (g)**	**Y4 (mJ)**	**Y5 (g)**	**Y6 (g)**	**Y7 (mm)**	**Y8 (MPa)**	**Y9 (%)**	**Y10 (MPa)**
DF	14.00	12.00	15.00	13.00	13.00	12.00	11.00	11.00	14.00	15.00
R^2	0.78	0.71	0.55	0.71	0.58	0.70	0.78	0.88	0.52	0.78
R^2 adj	0.73	0.59	0.49	0.62	0.45	0.58	0.66	0.82	0.42	0.75
Q^2	0.73	0.53	0.39	0.48	0.31	0.42	0.47	0.68	0.32	0.75
Model terms	4.00	6.00	3.00	5.00	5.00	6.00	7.00	7.00	4.00	3.00
DF residual	14.00	12.00	15.00	13.00	13.00	12.00	11.00	11.00	14.00	15.00
RSD	0.02	0.15	13.85	0.04	1333.50	0.17	5.44	4.58	40.25	0.28
p model	0.00	0.00	0.00	0.00	0.02	0.01	0.00	0.00	0.01	0.00
DF lack of fit	12.00	10	13	11	11	10	9	9	12	13
p lack of fit	0.11	0.06	0.07	0.32	0.43	0.03	0.44	0.08	0.25	0.19
SD pure error	0.01	0.04	4.01	0.02	1055.51	0.03	4.44	1.51	22.95	0.14

Y1 - Film thickness (mm), Y2 - Swelling degree (%), Y3 – Adhesive force (g), Y4 – Adhesiveness (mJ) Y5 – Hardness (g), Y6 - Rigidity at 5 mm (g), Y7 - Deformation at target (mm), Y8 - Tensile strength (MPa), Y9 - Elongation at break (%), Y10 - Young's modulus (MPa).

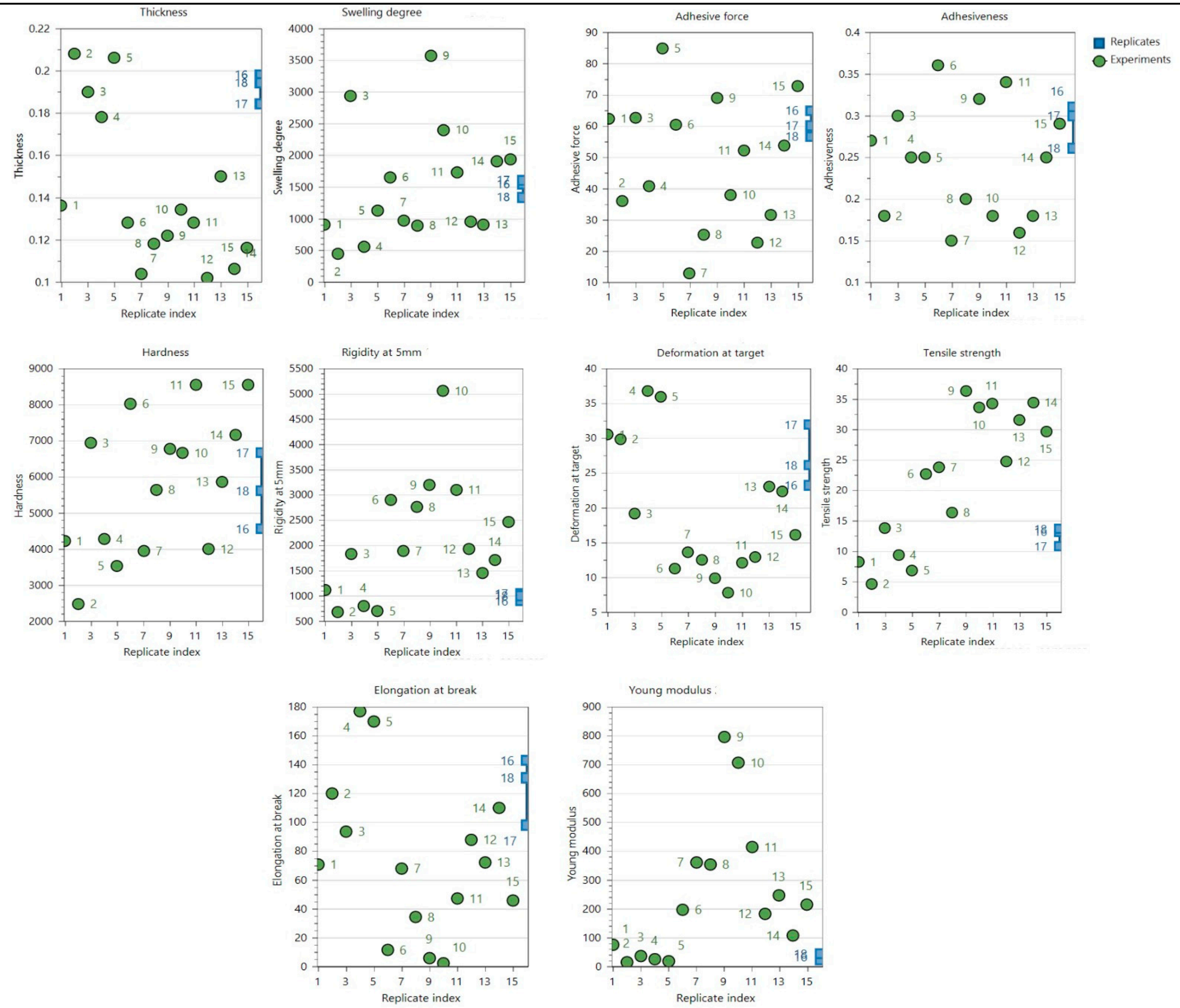

Figure S2. Replicate plots.

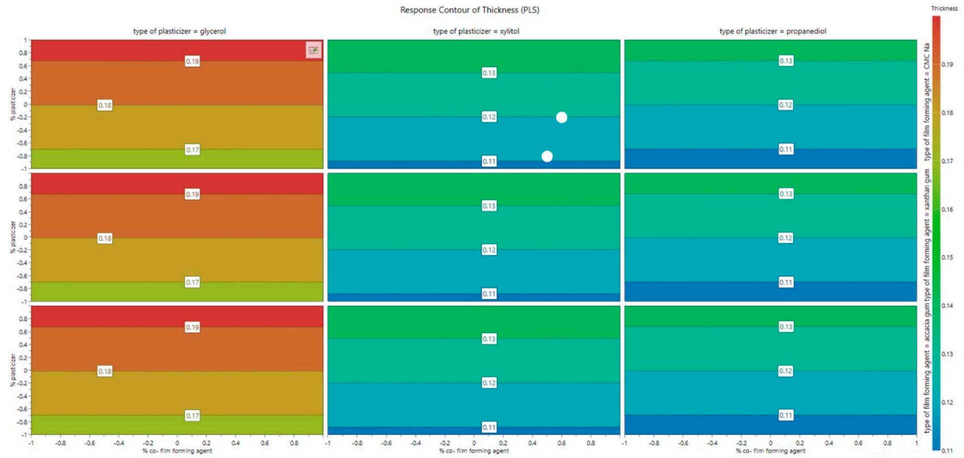

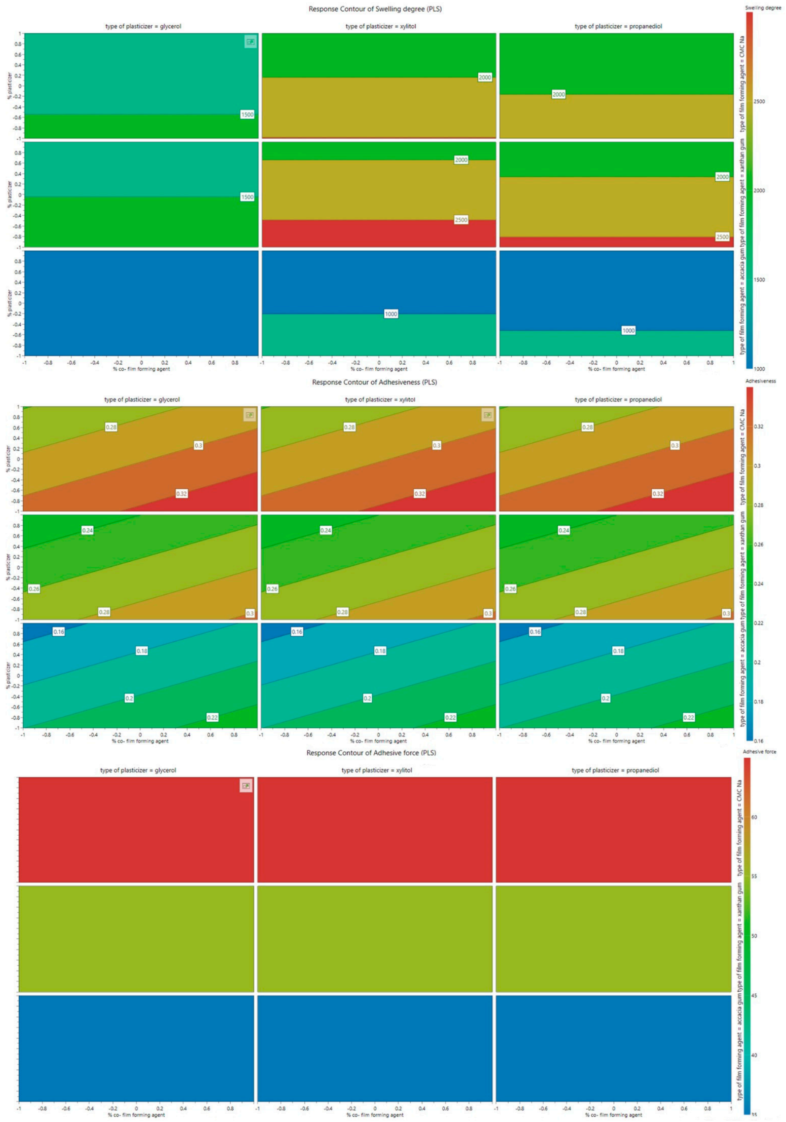

Figure S3. Contour 4D plots (part 1/2).

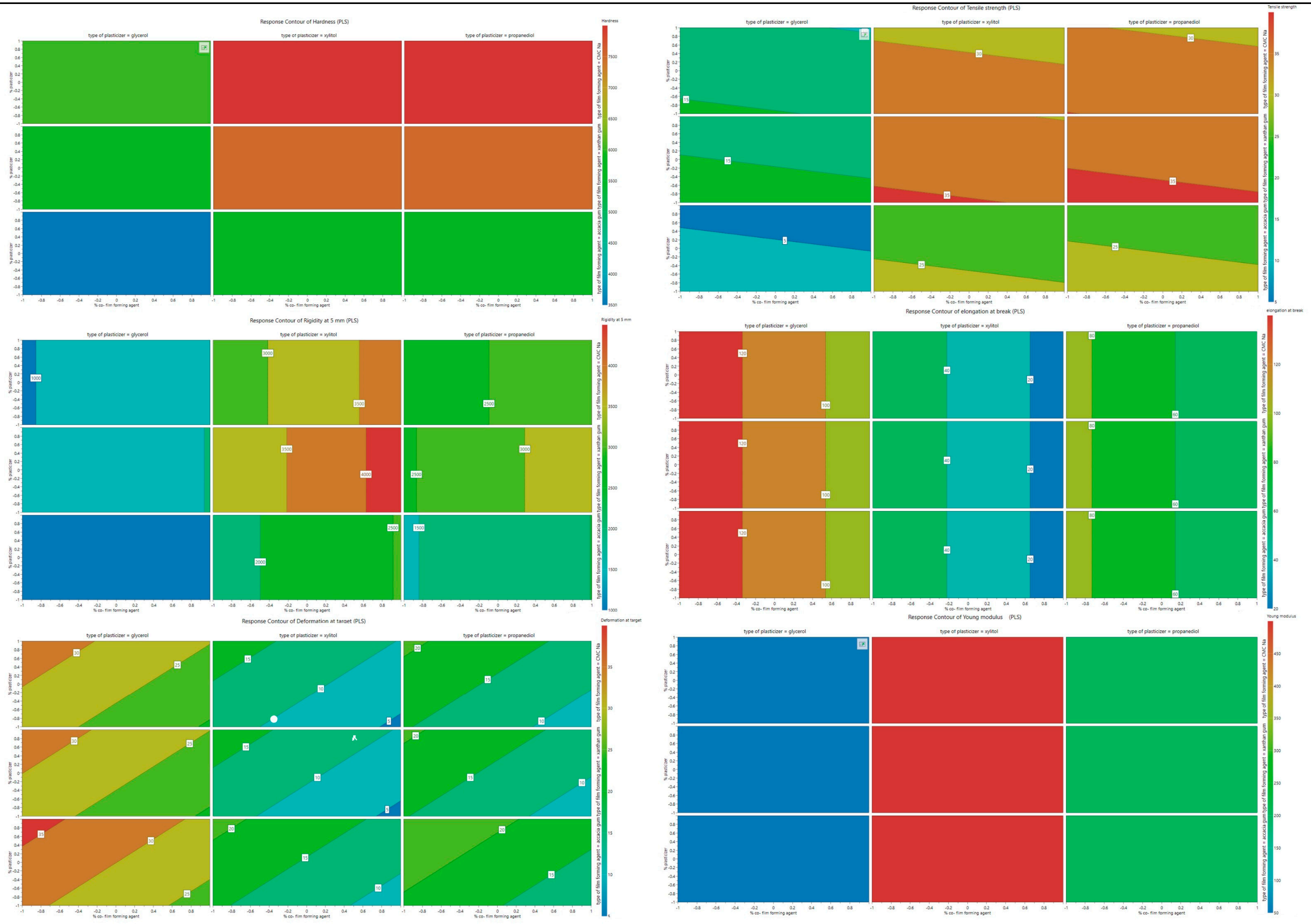

Figure S3. Contour 4D plots -part 2/2.
